# Factors associated with modern contraceptives use among postpartum women in Bukombe district, Geita region, Tanzania

**DOI:** 10.1371/journal.pone.0239903

**Published:** 2020-10-14

**Authors:** Michael Johnson Mahande, Emmanuel Shayo, Caroline Amour, Gerry Mshana, Sia Msuya

**Affiliations:** 1 Kilimanjaro Christian Medical University College, Moshi, Tanzania; 2 Department of Epidemiology & Biostatistics, Institute of Public Health, Kilimanjaro Christian Medical University College, Moshi, Tanzania; 3 National Institute of Medical Research, MITU- Mwanza, Mwanza, Tanzania; 4 Department of Community Health, Institute of Public Health, Kilimanjaro Christian Medical Centre, Moshi, Tanzania; University of Michigan, UNITED STATES

## Abstract

**Introduction:**

Modern contraceptive use during the first year postpartum potentially prevents unplanned pregnancies and help to improve maternal and child health. Therefore, identifying factors associated with contraceptive utilization among women of reproductive age during extended postpartum period is essential.

**Objective:**

This study aimed to assess factors associated with modern contraceptives use among postpartum women in Bukombe District, Geita region.

**Method:**

A community-based cross-sectional study was conducted among women who were in their first year after child birth in Bukombe district. A total of 511 women were included using multistage sampling techniques. Data were collected using a structured questionnaire. Data analysis was performed using Stata 15 (College Station, Texas, USA).

**Results:**

The prevalence of postpartum modern contraceptive was 11.9%. The most frequently used method was implant (6.5%). Most women started to use the contraceptive during the first three months after delivery. Living in urban (AOR = 1.85, 95% CI: 1.20–3.79), having business (AOR = 2.35, 95% CI: 1.31–2.28), last born aged 3–4 months (AOR = 3.31, 95% CI: 1.11–9.85) and menses resumption (AOR = 9.24, 95% CI: 3.60–23.72) were predictors for postpartum contraceptive use. However, fear of side effects, poor knowledge about contraceptives, husband restrictions, distance to health facility and contraceptive availability were reported as barriers for postpartum modern contraceptive use.

**Conclusion:**

Prevalence of postpartum modern contraceptive use in the study area is still low. Numerous factors were reported as barriers for postpartum contraceptive use. A strategy such as health education on befits of post-partum modern contraceptive use and counseling women about side effects may help to improve its uptake.

## Introduction

Postpartum family planning (PPFP) is defined as the prevention of unintended pregnancy and closely spaced pregnancies through the first 12 months following childbirth [1]. Most countries in sub-Saharan Africa are characterized by high fertility rates as well as population growth. It has been estimated that most countries will grow by 100–300 percent by 2050 and the region population will double over the next 45 years [2]. The main driver of high fertility rate in most African countries is persistent demand for large numbers of children [1]. Fertility rate would decline only if women will be provided with greater access to quality family planning services as a response to unmet needs for modern contraceptives, especially during the post-partum period [3].

Although some progress has been made in terms of increasing women’s access to maternal and child care services in Sub-Saharan Africa, no significant improvement has been observed in contraceptives use among postpartum mothers within the first year of delivery [4]. Previous studies have demonstrated that use of postpartum modern contraceptives is very low in the region [5–9]. In sub-Saharan Africa, pregnancies within the first 12 months after delivery are more likely to end up with unsafe abortion due to poor access to family planning during the postpartum period [10]. Thus, high levels of unplanned pregnancies in the first year postpartum expose women to risk of death due to a lack of safe abortion services. Poor utilization of modern contraceptive may also lead to short interpregnancy interval which has been associated with an increased maternal morbidity such as anemia, bleeding disorders, premature rupture of membranes, puerperal endometritis and mortality [11]. These serious problems could be avoided by the use of an effective family planning methods within the immediate or extended postpartum period. In the developing world, spacing pregnancies for at least two years apart have been reported to reduce by more than 40% and 31% for maternal and under five mortality respectively [10].

Therefore, reducing maternal deaths, prevention of unintended and too-early pregnancies is of public health priority. Previous authors have demonstrated that, maternal and child mortality can be prevented by 30% and 10%, respectively if couples could use modern contraceptives to space their pregnancies for at least two years apart [12]. This can be scaled up through counseling on contraceptive methods from early in pregnancy and throughout postpartum period [13, 14].

Numerous factors have been associated with poor utilization of postpartum modern contraceptives. These include desire to conceive in the near future, fear of side effects, lack of freedom to stop the method without involving the health provider, lack of knowledge about the method and method availability [6, 15–17]. Furthermore, misconceptions, accessibility of the method, limited skills among health care providers in modern contraceptive insertions [17–19], concerns about side effects and the effectiveness of the methods in preventing pregnancy [20, 21], also have been reported as barriers for the modern contraceptives use.

The government of Tanzania has made efforts to ensure that family planning services are integrated into the reproductive and child health (RCH) services. The National Family Planning Costed Implementation Program (NFPCIP-2009) plan was set to identify resources and actions required to make family planning services accessible to all citizens in order to achieve at least 60% of contraceptive prevalence by 2015 [22]. However, this national target was not met. The nation’s contraception prevalence rate remains as low as 32% and 46% for married and unmarried women respectively [8]. Furthermore, the Ministry of Health and Social Welfare also updated the National Family Planning Research Agenda (NFPRA) as an attempt aimed at identifying current gaps in family planning through evidence-based knowledge [22]. Further commitments are included in One Plan II which targets to achieve a national modern contraceptive prevalence rate (mCPR) of 45% by 2020 and reduce the unmet need of family planning (FP) to 10% by 2020 [23]. The Tanzanian government has also targeted to double the number of FP users to 4.2 million by 2020 as part of FP2020 initiatives [24].

Despite these efforts to ensure that contraceptive uptake is optimized during post-partum period in Tanzania, little is known about the factors associated with use of modern contraceptives during post-partum period. This study aims at determining factors associated with uptake of modern contraceptive among postpartum women in Bukombe District, Geita region.

## Methodology

### Study design and setting

This study was a community based cross-sectional study design that was conducted in May–June 2018, using both quantitative and qualitative methods. The study was conducted in a rural pastoral community of Bukombe district in Geita region in north-western Tanzania. Bukombe district is among of the 5 districts of Geita region which is located in the lake zone. It has a population of 224,542, 13 wards; 122 villages, an average of house hold size of 5.9 and fertility rate of 5.5 [8]. Geita is one of the regions with poorest indicators with regards to maternal and new born health in Tanzania. Businesses, small farming and mining are the main activities in the region.

### Study population and sampling technique

All women of reproductive aged between 15–49 years were included. We excluded women who were non-residents in past 6 months and those who had hysterectomy. Multistage sampling process was used to get respondents. The first stage involved purposive the selection of two divisions out of three divisions in Bukombe district. The second stage involved selection of 3 out of 17 wards. Proportionate to size sampling guided selection of wards from each division. The third stage involved random selection of three villages from each of the selected wards where 9 villages were selected. At each village three hamlets (is a sub village or the smallest administrative unit area) were randomly selected and women of reproductive age who gave birth in past one year from the selected hamlets were identified by local leaders through door to door approach, and those who were eligible were invited to participate. The final sample size comprised of 511 women.

### Data collection method and tool

A standardized questionnaire in Kiswahili language which was adopted from the Tanzania Demographic Health Survey with slight modifications to include cultural and belief questions (i.e. fertility issues) was used to collect data from the study participants through face to face interview. The information collected include: social demographic characteristics, children and reproductive health history and contraceptive methods availability, use and preferences. Seven research assistants employed to assist the collection of data. Research assistants received one day training session involving briefing on the purpose of the study, meaning of terms used in the study and the importance of maintaining ethical standards during data collection process.

Before the interview the researchers explained the objectives of the study to the participants and request her to participate. The participants signed the consent form if she accepts and researcher continue to ask the questions. All issues related to privacy and confidentiality were adhered. Face to face interviews were conducted in a private secluded area in order to maintain confidentiality.

### Study variables

The main outcome variable was current use of postpartum modern contraceptives. The independent variables include; socio-demographic variables such as religion, marital status, education level, employment status and partner age difference. Reproductive health variables such as parity, live children, age of the last born, desired number of children, desired birth interval, mode of delivery and place of last delivery were also explored. Information on challenges regarding postpartum family planning use were also sought from the study participants using open ended questions. These open-ended questions where later on coded and quantified into the different themes that emerged.

### Ethical consideration

Ethical clearance was obtained from Kilimanjaro Christian Medical University College Research Ethics Committee. Permission to carry out the study was obtained from Geita region and Bukombe district administrative authorities. Written consent was obtained from every participant. Anonymity was maintained by using unique identifiers instead of names to maintain confidentiality. Participation was voluntary and the participant’s right to withdraw from the study without giving any reason was explained.

### Statistical analysis

Data were analyzed using STATA software, version 15. Continuous variables summarized by measure of central tendency and their respective dispersion. Categorical variables were summarized by proportions and frequencies. Odds ratio and 95% confidence interval used for factors associated with modern contraceptive use among postpartum women were estimated in multivariable logistic regression model. A p-value of less than 5% was considered significant.

## Results

### Socio-demographic characteristics of the study participants

A total of 511 women of reproductive age were studied. This corresponds to response rate of 100%. Majority 361 (70.7%) of the study women were aged between 26 to 34 years. The mean (SD) age was 26.48 (6.79) years. Similarly, 241 (60.4%) participants’ partners were aged between 26 to 40 years with their mean (SD) age of 33.9 (6.9) years. Majority 273 (68.4%) of these partners had primary education, where 258 (64.7%) were doing farming activities (Table 1).

**Table 1:** Socio-demographic characteristics of the study participants (N = 511).

Characteristics	n	%
Age		
15–24	72	14.1
24–34	361	70.7
35+	78	15.2
Religion		
No Religion	86	16.8
Christian	403	78.9
Muslim	22	4.3
Marital Status		
Single/Not in union	74	14.5
Married/Cohabiting	437	85.5
Education		
No Formal	171	33.5
Primary	310	60.6
Secondary +	30	5.9
Occupation		
None	96	18.8
Laborer	22	4.3
Business	89	17.4
Farming	304	59.5
Residence		
Rural	340	66.5
Urban	171	33.5
Income Level (Tshs)		
Low <100000	415	81.2
Normal ≥ 100000	96	18.8
Partner Age (years)		
< = 25	100	25.1
26–40	241	60.4
> = 41	58	14.5
Partner’s Education level		
No Formal education	74	18.5
Primary	273	68.4
Secondary and above	52	13.1
Partner’s Occupation		
Formal employed	13	3.2
Daily worker/laborers	47	11.8
Business	75	18.8
Farming	258	64.7
Driver	6	1.5
Partner has other wives		
No	307	76.9
Yes	92	23.1

### Reproductive health related characteristics of study participants

Majority 379 (76.4%) of the participants expressed to have more than five children. Most 307 (72.2%) of the women wanted to delay their next birth by at least 3 years. A greater number, 384 (75.1%) of the respondents reported to have not resumed menstruation at the time of the study. More than half 286 (56%) reported ever use of modern contraceptive (Table 2).

**Table 2:** Reproductive health related characteristics of study participants (N = 511).

Characteristics	n	%
Parity		
≤2	208	40.7
3–4	147	28.8
≥5	156	30.5
Number of live children		
≤2	233	45.6
3–4	149	29.2
≥5	129	25.2
Age of last born (months)		
≤3	162	31.7
4–6	136	26.6
7–12	213	41.7
Desired number of children (n = 496)		
≤2	14	2.8
3–4	103	20.8
≥5	379	76.4
Desired birth interval (n = 425)		
≤2	118	27.8
≥3	307	72.2
Mode of delivery		
Vaginal	493	96.48
Caesarean	18	2.94
Place of delivery		
Home	249	48.7
Dispensary/Health Centre	134	26.2
Hospital	128	25.1
Ever use family planning		
No	286	56.0
Yes	225	44.0
Ever heard family planning		
No	14	2.7
Yes	497	97.3
Resumption to menstruation		
No	384	75.1
Yes	127	24.9

### Prevalence of postpartum modern contraceptive use

**Fig 1:**
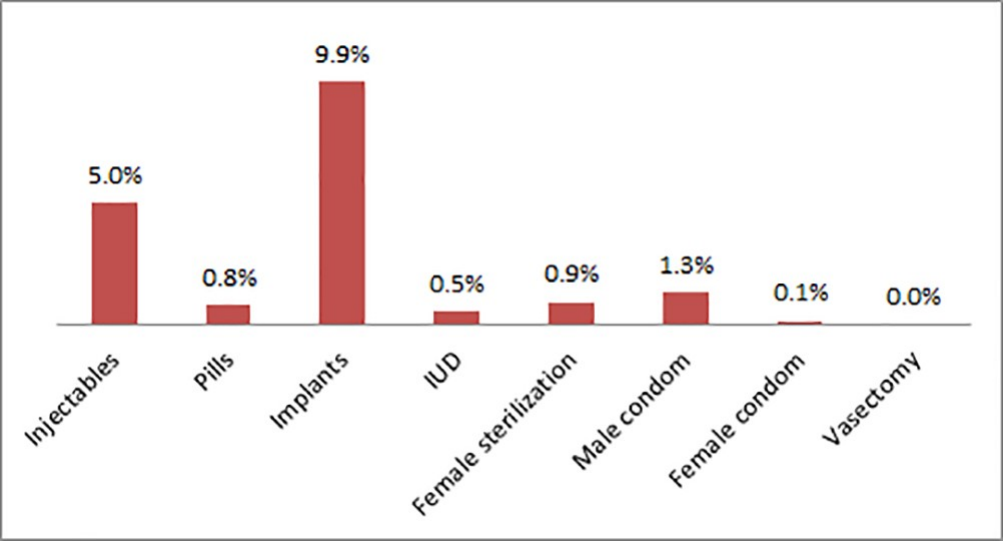
The most preferred contraceptive method among postpartum women (N = 61).

The proportion of post-natal mothers who reported using postpartum modern contraceptives was 61 (11.9%). The most frequently reported modern contraceptives were implant (6.5%) and injectable (3.5%) while pills (0.9%), female sterilization (0.5%), intra uterine device and male condoms (0.2%) were infrequently reported (Fig 1).

### Time to contraceptive use after delivery among postpartum women

**Fig 2:**
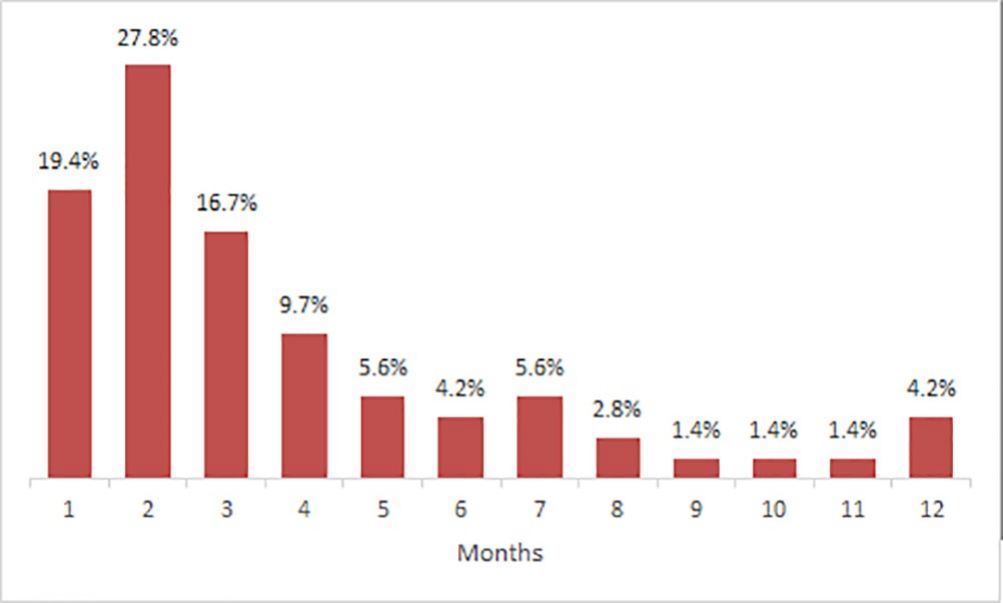
Time to contraceptive use after delivery (N = 61).

Majority of post-natal mothers started using the contraceptive methods after one month to three months post-delivery. The highest uptake occurred at the second month post-delivery (33%) while the proportion of mother who started using contraceptive from the fourth month up to the seventh were nearly the same. The lowest uptake occurred from the eight month to eleventh month of age (Fig 2).

### Socio-demographic and reproductive factors associated with postpartum modern contraceptive use

In bivariate analysis, urban area of residence (COR = 1.859, 95% CI: 1.21–3.41) and business women (COR = 2.095, 95% CI: 1.38–3.41) were significantly associated with postpartum contraceptive use. This association also remained significant in multivariable analysis where business women (AOR = 2.348, 95% CI: 1.31–2.28) and urban area of residence (AOR = 1.846, 95% CI: 1.20–3.79) were significant associated with postpartum modern contraceptive use. Age of last born, desired birth interval and resumption of menstrual were significantly associated with post-partum contraceptive use in bivariate analysis. After adjustment, last born aged 3–4 months (AOR = 3.307, 95% CI: 1.11–9.85) and resumption of menstrual (AOR = 9.236, 95% CI: 3.60–2.72) were significantly associated postpartum modern contraceptive use. Furthermore, husband restrictions, lack of awareness on importance of postpartum contraceptives, longer distance to health facilities and poor availability of postpartum contraceptive methods were associated with lower odds of postpartum modern contraceptives usage (Table 3).

**Table 3:** Socio-demographic characteristics associated with postpartum modern contraceptive use (N = 511).

Variables	Users N (%)	COR (95%CI)	P-value	AOR (95% CI)	P-value
Age					
15–24	3(4.1)	1			
25–34	48(13.3)	1.16(0.30–4.58)	0.828	1.65 (0.55–4.93)	0.370
35+	10(12.9)	0.833(0.19–3.75)	0.812	2.05 (0.60–7.05)	0.255
Religion					
No Religion	5(8.8)	1			
Christian	45(12.7)	1.186(0.41–3.43)	0.753	1.044 (0.44–2.46)	0.921
Muslim	11(50.0)	2.5(0.52–11.89)	0.250	1.836 (0.65–5.22)	0.255
Marital Status					
Single/Not in union	17(23.0)	1			
Married/Cohabiting	44(10.0)	0.543(0.27–1.09)	0.088	0.808 (0.52–1.25)	0.339
Education					
No Formal	18(10.5)	1			
Primary	39(12.6)	0.849(0.43–1.66)	0.633	0.742(0.48–1.15)	0.179
Secondary and above	4(13.3)	0.563(0.16–1.94)	0.363	0.475(0.18–1.24)	0.129
Occupation					
None	8(8.3)	1			
Daily worker	5(22.7)	2.422(0.62–9.45)	0.103	2.713 (1.26–5.83)	0.011
Business	20(22.4)	2.095(1.38–3.41)	0.011	2.326 (1.16–4.65)	0.017
Farming	28(9.2)	1.466(0.60–3.57)	0.400	1.964 (0.99–3.90)	0.054
Income Level (Tshs)					
Low <100000	44(10.6)	1.36(0.34–1.58)	0.521	1	
Normal ≥ 100000	17(17.7)	0.43(0.19–2.75)	0.212	0.620 (0.39–0.98)	0.042
Residence					
Rural	24(7.1)	1		1	
Urban	37(21.6)	1.859(1.21–3.41)	0.025	1.618 (1.02–2.57)	0.041
Parity					
≤2	22(10.58)	1		1	
3–4	22(14.97)	1.089(0.53–2.23)	0.816	1.065 (0.69–1.65)	0.779
≥5	17(10.90)	0.676(0.32–1.42)	0.300	0.574 (0.32–1.02)	0.059
No. Children Alive					
≤2	25(10.73)	1		1	
3–4	21(14.09)	0.98(0.49–1.97)	0.955	-	
≥5	15(11.63)	0.730(0.35–1.55)	0.411	-	
Age of last born(months)					
≤3	15(9.26)	1		1	
4–6	23(16.91)	2.345(1.07–5.12)	0.032	1.979 (1.17–3.36)	0.011
7–12	23(10.80)	1.246(0.59–2.63)	0.564	1.169 (0.69–1.97)	0.557
Desired no. children					
≤2	2(14.29)	1		1	
3–4	14(13.59)	0.757(0.12–4.60)	0.762	0.440 (0.08–2.38)	0.341
≥5	45(10.82)	0.781(0.14–4.43)	0.780	0.425 (0.08–2.16)	0.302
Desired Birth Interval					
≤2	1(0.2)	1		1	
≥3	60(14.1)	9.665(1.25–74.63)	0.030	2.076(0.51–7.75)	0.318
Mode of Delivery					
Vaginal	59(11.97)	1		1	
Caesarean	2(13.33)	0.814(0.16–4.15)	0.804	1.009 (0.29–3.52)	0.988
Place of Delivery					
Home	17(6.83)	1		1	
Disp/HC	19(14.50)	1.332(0.62–2.85)	0.460	1.088 (0.56–2.12)	0.805
Hospital	25(19.53)	1.752(0.85–3.63)	0.131	1.233 (0.62–2.45)	0.549
Resumption menstruation					
No	19(3.76)	1		1	
Yes	42(8.2)	5.558(2.61–11.82)	<0.001	3.051 (1.58–5.88)	0.001
Barriers to contraceptive use					
Fear of side effects	31 (50.8)	2.2 (0.7–6.6)	0.159	1.9 (0.6–6.1)	0.259
Husband restrictions	11 (18.03)	0.9 (0.3–3.1)	0.913	0.9 (0.3–3.2)	0.917
Lack of awareness	7 (11.5)	1.0 (0.3–3.8)	0.959	0.9 (0.2–3.6)	0.912
Distance to facility	3 (4.9)	0.5 (0.1–2.6)	0.439	0.7 (0.1–3.7)	0.668
Availability of methods	4 (6.6)	4.5 (0.8–27.4)	0.100	5.7 (0.9–37.2)	0.067
No challenge	5 (8.2)	1		1	

### Challenges related to postpartum modern contraceptive use

**Fig 3:**
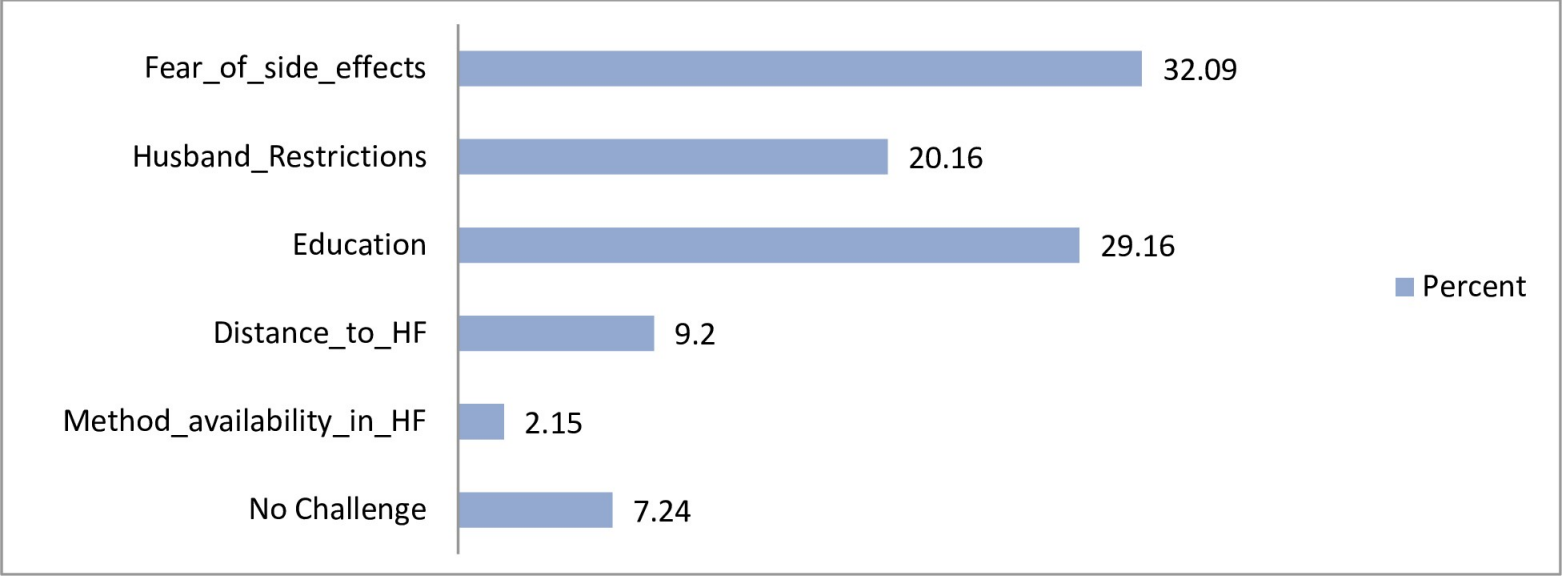
Challenges of postpartum modern contraceptive use.

Participants were asked regarding their views on challenges related to postpartum modern contraceptive use. A number of challenges were reported to hinder use of post-partum modern contraceptives. These include fear of side effects (32.1%), lack of awareness of contraceptives (29.1%), husband restrictions for their wives to use contraceptives (20.2%), distance to health facility (9.2%) and unavailability of the method in the health facility (2.1%) (Fig 3).

## Discussion

Results from the study showed that the proportion of contraceptive use among postpartum women is still low. Only 11.9% of postpartum women reported using a modern method of contraception at the time of the survey. Factors such as mother’s occupation, area of residence, age of last born and resumption of menstrual were significantly associated with postpartum contraceptive use.

The prevalence of modern contraceptive use in our study is lower than 32% for the national prevalence [8]. The lower prevalence of post-partum contraceptives use in the study area could be explained by high proportion of home deliveries (61%), coupled with poor attendance to postnatal care in the first 2 days (13%) in the district [8]. These leads to missed opportunities for postnatal contraceptive counseling. The post-partum contraceptive use in the present study is also lower than the prevalence of postpartum contraceptive (28%) in Uganda [4]. This may be due to the fact that the former study was done among women in the postpartum period, but were highly motivated to use family planning methods through a series of seminars. The findings suggest that facility delivery remain important windows of opportunity to provide access to family planning messages and to offer women various contraceptive methods. In the present study, the highest proportion of postnatal mothers uses implant contraceptives. This might be due to the fact that implant is not user dependent and may not easily seen by partners [11, 25]. This may again partly explain the partners’ influence over the women’s contraceptive choice [26, 27]. Our finding is in contrast with studies in Uganda and Malawi which showed injectable was the most preferable contraceptive [4, 21, 28]. The difference of these findings may be explained by difference in social cultural factors between the two populations. This suggests the need for more emphasize in empowering women during contraceptives counseling, to freely choose a family planning method.

In this study we have found that, women’s occupation was a significant factor influencing use modern contraceptives. Mothers who owned business had 2 times higher odds of using the contraceptives compared to those in other occupation such as famers and daily walkers. The probable explanation for observed association could be that, mothers who are involved in business activities make an interaction with many people who could have acquired the necessary knowledge to empower them with positive attitude towards the contraceptive use compared to others. This finding is consistence with previous study in Ethiopia by [29]. The similarities in finding could be due to social cultural factors and the study settings as these studies were conducted among urban residents and rural residents.

We found a significant difference in contraceptive use between Urban and Rural dwellers. Women residing in urban two-fold higher odds of using post-partum contraceptives compared to rural dwellers counterparts. The studies done in Awassa and Bahirdar in Ethiopia support this finding [1, 30]. The possible explanation could be that urban women have better access to information, education and health facilities than rural women.

Women whose menses had resumed after birth had 9-fold higher odds of using post-partum modern contraceptive compared to women whose menses have not returned (experiences amenorrhea). This finding could be justified by the fact that women may be aware of fertility return when their menses have resumed. Amenohorric women would perceive that they are less likely to get pregnant, by assuming that amenorrhea would protect against pregnancy irrespective of the postpartum duration. Similar finding was reported in Kenya [31].

The present study also showed that a woman with a child aged four to six months had higher odds of using contraceptives compared to women with children of other ages. The probable explanation to the observed association could be that most of mothers started to experience their menses at the third and fourth month after delivery.

This study found that fear of side effects, poor knowledge about contraceptives, husband restrictions in using contraceptives were the most hindering factors for post-partum contraceptive use. In addition, distance to health facility and availability of the method were among the challenges to use postpartum contraception. This finding is consistent with previous studies [32–35]. The fear of side effect might be based on their personal experiences or those other women they know or simply on unfounded perception. Some of the women attribute changes in their menstrual cycle to development of diseases in the reproductive system such as uterine fibroids. Prolonged and irregular vaginal bleeding has serious socio-cultural implication for many women. Some modern contraceptives methods such as Implant has been associated with prolonged bleeding after its removal, where some women experience changes in menstrual bleeding patterns [36]. This may be one of the possible reasons explaining the fear associated with postpartum modern contraceptive usage.

### Study limitations

The sensitive nature with issues of sex and contraceptive use could be a limitation as respondents may be reluctant to provide certain information, they consider to be intimate. This potential limitation was addressed by assuring the participants that their responses could not be traced to them but only used for academic purposes. This assurance might have encouraged majority of them to provide answers that actually reflect their reproductive behavior. The cross-sectional design of this study limited the ability to understand patterns of use or non-use across individuals over time.

### Conclusions

The postpartum modern contraceptive use in the study area is still low as 12%. The most preferred contraceptive was implant. Majority of the women had started using contraceptives at the first three months after delivery. Occupation, place of residence, age of the last born and menses resumption were associated with modern contraceptive use. Fear of side effects was the most prevalent challenge on contraceptive use.

Adequate counseling on modern contraceptive use (including side effects) during antenatal care, immediate post-delivery and throughout post-partum period is warranted. Provision of better access to information, education and health facilities in rural area may improve uptake of postpartum contraceptives.

## Supporting information

S1 Data (DTA)
